# Evaluation of a youth-focused suicide prevention HOPE aftercare service: protocol for a non-randomized hybrid effectiveness-implementation type I design

**DOI:** 10.1186/s12913-024-11795-2

**Published:** 2024-11-13

**Authors:** Dzenana Kartal, Tess Jaeger, Michelle Lamblin, Hannah Richards, Katrina Witt, Jo-An Occhipinti, Cathrine Mihalopoulos, Mary Lou Chatterton, Andrew Chanen, Patrick McGorry, Adam Skinner, Isabel Zbukvic, Andrew Thompson, Jonathan Knott, Anna Flego, Craig Hamilton, Marianne Webb, Edward Mullen, Natasha Swingler, Bridget Kenny, Jo Robinson

**Affiliations:** 1 Orygen, 35 Poplar Road, Parkville, 3052 Australia; 2 Centre for Youth Mental Health, The University of Melbourne, Parkville, 3010 Australia; 3 Brain and Mind Centre, University of Sydney, 94-100 Mallett Street, Sydney, 2050 Australia; 4 Computer Simulation & Advanced Research Technologies (CSART), 36 Paddington Street, Paddington, 2021 Australia; 5 School of Public Health and Preventive Medicine, Monash University, 553 St Kilda Road, Melbourne, 3004 Australia; 6 Warwick Medical School, University of Warwick, Coventry, CV4 7AL UK; 7 Department of Critical Care, The University of Melbourne, Parkville, 3010 Australia; 8 School of Population Health, The University of Melbourne, Parkville, 3010 Australia; 9 Royal Children’s Hospital, 50 Flemington Road, Parkville, 3052 Australia; 10 Centre for Mental Health Nursing, University of Melbourne, Barry Street, Carlton, 3053 Australia

**Keywords:** Suicide prevention, Aftercare, Young people, Effectiveness, Implementation, Program evaluation

## Abstract

**Background:**

Suicide is the fourth leading cause of death among young people aged 15–29 worldwide. Young people often present to emergency departments (EDs) with self-harm and suicide-related behaviors. The period following discharge from the ED is recognized as one of elevated risk for both repeated self-harm and suicide. During this critical time, suicide prevention aftercare services are recommended. Despite their increased popularity, evidence demonstrating the effectiveness of these models is very limited.

**Methods:**

Using a hybrid effectiveness-implementation type I design, this evaluation will assess the effectiveness and implementation of a suicide prevention aftercare (*Hospital Outreach Post-suicidal Engagement; HOPE*) service designed to reduce risk of self-harm and suicide in young people aged 12–25 who are referred to the service following an ED presentation for self-harm or suicide attempt. Two complementing theoretical frameworks will guide this evaluation, specifically the design, data collection, analysis, and interpretation of results. The RE-AIM evaluation framework will be used to assess *Reach*, *Effectiveness* (including cost-effectiveness), *Adoption*, *Implementation* and *Maintenance* of the HOPE aftercare service. The PRISM implementation framework will be used to assess multi-level contextual factors hypothesized to affect the RE-AIM outcomes. Several data sources will be used to assess the changes in primary and secondary outcomes from baseline to post–intervention, and at follow-up, including user and provider self-report surveys, semi-structured interviews, and routinely collected hospital data. An historical control study will also be conducted using data from the Self-Harm Monitoring System for Victoria to examine the impact of the service on rates of self-harm and suicide-related presentations to ED, and compare trends prior to and following commencement of the HOPE aftercare service. In addition, dynamic systems modelling will be used to assess the future scalability of the service.

**Discussion:**

Findings from this evaluation will determine the effectiveness, including cost-effectiveness, of the HOPE aftercare service and describe the implementation context. They will inform the future development and sustainability of this and other similar services across Australia and internationally.

**Trial registration:**

This trial was prospectively registered with the Australian New Zealand Clinical Trials Registry (ANZCTR) on the 19th December 2023 (Registration number ACTRN12623001332617). We do not foresee any amendments to this protocol however, if any unforeseen modifications are required, they will be submitted to ANZCTR.

**Trial sponsor:**

Orygen, 35 Poplar Road, Parkville, VIC, 3052, Australia.

## Background

### Rates of suicide

Suicide is the fourth leading cause of death among young people aged 15–29 worldwide [1] and the leading cause of death among young Australians [2]. In 2022, deaths by suicide represented 30.9% of all deaths in young Australians aged 15–17 and 32.4% of all deaths in those aged 18–24 years, up from 16.5% to 23.9% respectively in 2001 [2]. For every young person who dies by suicide, many more engage in self-harm, and more still live with suicidal ideation [3]. Both suicide ideation and self-harm (i.e., intentional drug overdose, self-injury and/or self-poisoning irrespective of motivation and degree of suicidal intent) [4] are the greatest predictors of future suicidal behavior [5, 6]. Whilst many young people do not seek help from services for self-harm, those who do often experience sub-optimal treatment responses [7, 8].

### Presentation rates

Many at-risk young people present to emergency departments (EDs) in crisis, following severe self-harm and/or a suicidal attempt [9]. In Australia, the hospital presentation rates are highest among young people aged 15–19 years (389 per 100,000 population) [10]. The rates of intentional self-harm hospitalization are steadily increasing [11]. The highest hospitalization rates are reported for females aged 15–19 and they have increased from 374 hospitalizations per 100,000 in 2008–09 to 637 hospitalizations per 100,000 in 2021–22 [10].

The risk of further suicide attempt/s is greatest immediately following discharge from the ED after a suicide attempt [7, 12] and remains high for up to 12 months following the attempt [12, 13]. Modelling studies with Australian data estimate that delivering better care at this time would reduce the numbers of self-harm hospitalizations and suicide deaths by 5.65% (95% CI, 4.87 − 6.42%) and 5.45% (4.68 − 6.22%), respectively [14]. This would mean ~ 1,616 fewer hospitalizations and ~ 33 fewer suicide deaths in Australia every year [15] .

### Aftercare service models

In response to this, aftercare services that provide support for people following their presentation to the ED with self-harm/suicidal ideation have received significant attention. Aftercare services typically aim to help an individual bridge the gap between hospital-based care during an acute crisis and establish ongoing support in the community [16]. They can be classified in three categories: brief contact interventions, brief interventions, and coordinated assertive aftercare [16]. Brief contact interventions are low-intensity and low-cost interventions such as supportive messages via postcard, text message or letter, that encourage engagement with services. Milner and colleagues’ [17] meta-analysis showed that brief contact interventions reduced frequency of suicide re-attempts and self-harm. Brief interventions are defined by a limited number of short sessions, with a significant proportion of sessions delivered via telephone, and focus on helping to understand the factors that lead to a suicidal crisis and help expand the individual’s coping strategies. The Attempted Suicide Short Intervention Program (ASSIP) [18] is an example of this approach that has been shown to reduce suicide re-attempts.

Coordinated assertive aftercare typically involves the following four components: (1) immediate follow-up post discharge from hospital, (2) ongoing risk assessment and planning, (3) motivational support to engage with treatment and (4) problem solving/solution-focused counselling [19]. Aftercare is offered over a defined period, usually 3–6 months. Currently there are several models of assertive aftercare globally that have demonstrated varying degrees of efficacy (e.g., Norwegian OPAC [Outreach, Problem Solving, Adherence, Continuity]) (e.g., 3), the AID model in Copenhagen [20], and the ACTION–J model in Japan [21].

In Australia, the Hospital Outreach Post-suicide Engagement (HOPE) aftercare for adults is the first such model implemented in the public healthcare system [22]. To date, there have been few evaluations of this model; an initial evaluation showed substantial improvements to suicidal ideation, distress, coping and wellbeing, among adults [23].

## Orygen’s child and youth-focused HOPE aftercare

Youth-focused HOPE aftercare has been developed based on the adult HOPE model [22]. Currently, there are four youth-specific services delivering HOPE aftercare in Victoria, Australia. The Orygen HOPE aftercare was co-designed in collaboration with young people and families with lived experience and is delivered by Orygen—a state-funded national center for youth mental health responsible for the provision of primary and secondary clinical services. Orygen provides clinical mental health services to young people aged 12–25 in the north–west metropolitan region of Melbourne—a large and diverse geographical area with high population growth. Of the 1.4 million residents, approximately 200,000 are aged between 15 and 25 [24].

Orygen HOPE aftercare has been operational since January 2022. To date, there has been no evaluation of this, or other youth-focused aftercare services, in Australia or internationally. The proposed evaluation will be the first to systematically evaluate an aftercare service for young people at high risk of suicide.

## Methods

### Study aims

The first aim of this evaluation is to assess the effectiveness of the youth-focused HOPE aftercare delivered by Orygen to reduce suicide-related behaviors and improve mental health outcomes among young people who have presented to ED following a significant self-harm event or a suicide attempt. Specifically, the primary objective is to evaluate the effectiveness of HOPE aftercare in reducing the frequency of suicidal ideation (primary outcome). The secondary objectives are to evaluate the impact of HOPE aftercare on hospital re-presentation rates, mental health outcomes, quality of life, and determine the cost-effectiveness and scalability of the intervention.

The second aim of this evaluation is to assess the implementation of the service. Specifically, to determine the feasibility of delivering the service, assess acceptability and satisfaction with HOPE aftercare, and identify contextual barriers and enablers.

### Evaluation frameworks

**Fig. 1:**
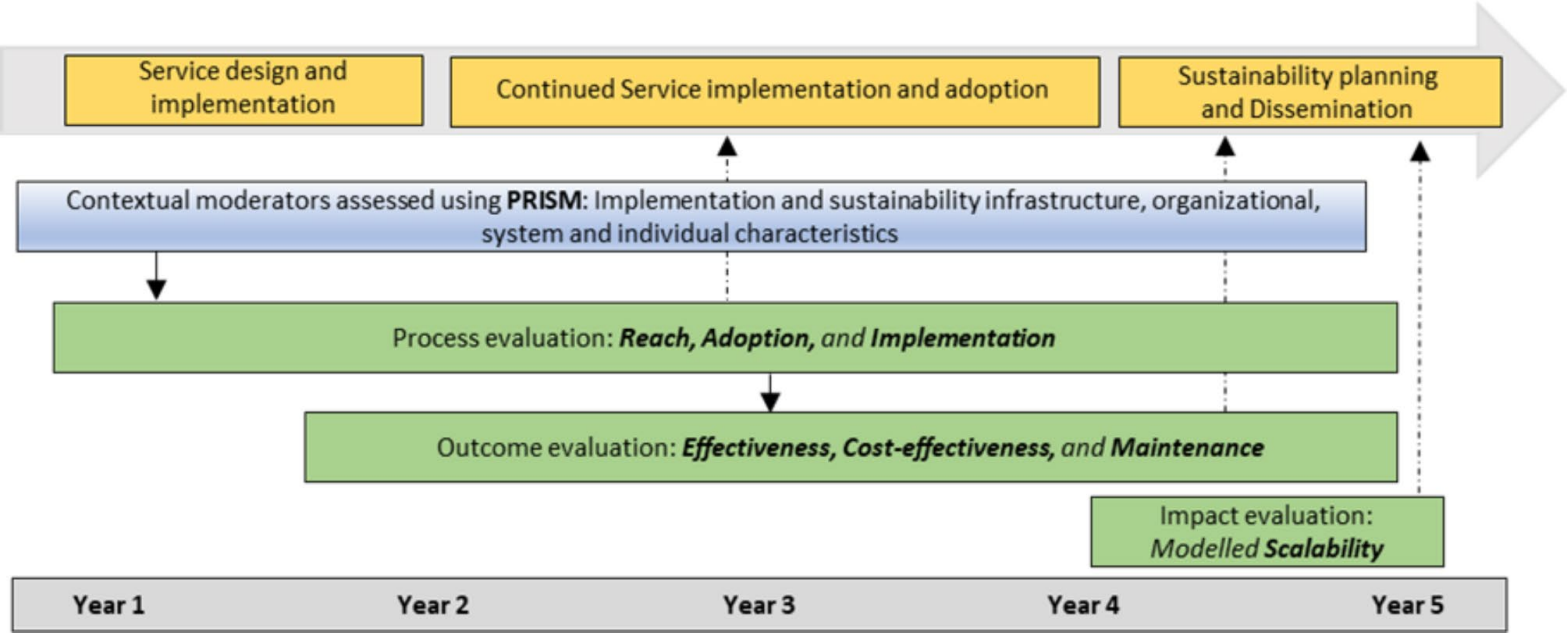
Overview of the project design illustrating the integration of RE-AIM and PRISM

Two complementing theoretical frameworks will be used to guide the evaluation: the Reach, Evaluation, Adoption, Implementation and Maintenance (RE-AIM) framework [25] and the Practical Robust Implementation and Sustainability Model (PRISM) framework [26], which is an extension of the RE-AIM framework. Specifically, to conceptualize the evaluation outcomes (i.e., service *Reach* and *Effectiveness*, including consideration of *Adoption* characteristics, *Implementation* process and the potential for long-term *Maintenance*) we will be guided by the RE-AIM framework. The PRISM framework will be used to systematically identify and assess multi-level contextual factors hypothesized to affect the RE-AIM outcomes. Specifically, this evaluation will assess how the perspectives of stakeholders including the service providers, the characteristics of recipients, the implementation and sustainability infrastructure of the organization, and the external environment, influence the evaluation outcomes. Furthermore, this evaluation was designed and will be delivered under the governance of a project steering group comprising academic, clinical and state department organization representatives and peer researchers with lived experience. Figure 1 provides an overview of the project design, illustrating the integration of the RE-AIM and PRISM frameworks.

### Study design

A mixed methods evaluation will be conducted over a five-year period. It will employ a type I hybrid effectiveness-implementation design [27], which focuses primarily on determining the effectiveness of an intervention, while exploring the context for implementation.

The RE-AIM and PRISM frameworks will inform all aspects of this evaluation, including development of the program logic map (see supplementary material 1), evaluation outcomes, selection of quantitative measures, and semi-structured interview guides. Table 1 provides a detailed overview of the study design, including evaluation domains and corresponding research questions, outcomes, operational definitions, measures, and data sources. This also provides a focal point for evaluation planning and supports the mapping of evaluation methods and associated data sources to program logic elements.

**Table 1:** Overview of study design, including domains of interest

**RE-AIM domain**	**Research Questions**	**Outcomes**	**Operationalization**	**Measures**	**Data Sources**									
					**Users**				**Providers**			**Systems**		
					**YP**		**Family**							
					Survey	Interview	Survey	Interview	Survey	Interview/ workshop	EOT form	ROM	Hospital	SHMS
Reach	Is the service reaching young people presenting with self-harm or suicidal ideation to ED in the catchment area? Who participates in the service and how do they participate over time?	*Participation (YP/Family)*	Proportions of service users presenting to ED who were eligible and who participated in HOPE aftercare	Routinely collected variables/data									X	
		*Engagement (YP/Family)*	Proportions of service users moving through the service including the referral, intake, retention and discharge points	Routinely collected variables/data									X	
		*Participant characteristics* *(YP/Family)*	Characteristics of service users (e.g., age, gender, location, priority group, mental health diagnosis, comorbidities, hospital presentations)	Routinely collected variables/data									X	
	What barriers/facilitators to participation exist?	*Barriers/Facilitators*	Identification of barriers and facilitators	Open-ended questions		X		X		X				
Adoption	How and when are ED staff referring young people to the service?	*Participation (Staff)*	Characteristics of the referral pathways	Routinely collected variables/data							X		X	
	What are the characteristics of the participating service system and health providers?	*Engagement (Staff)*	Characteristics of providers (e.g., age, gender, years of service, professional background, roles)	Demographic variables					X					
		*Service settingcharacteristics*	Characteristics of the service setting (e.g., organizational climate and culture, service resources, leadership capacity, staff integration)	CFIR–IS ILS PSI					X					
		*Provider characteristics*	Characteristics of providers’ training, knowledge, self-efficacy and attitudes/perceptions about suicide	Purpose-developed items based on existing measures. ASP scale					X					
	What barriers/ facilitators exist, and how do they affect the program access, engagement and efficacy?	*Barriers/Facilitators*	Identification of barriers and facilitators	Open-ended questions		X		X		X				
Implementation	What are the contextual factors (i.e., organizational, system and individual) impacting implementation?	*Moderators*	Descriptors of changes or modifications to the intervention							X	X			
	How satisfied are young people/family members with the service?	*Satisfaction*	User satisfaction rates	Routinely collected data Purpose-developed questions		X		X	X	X		X		
	What level of delivery is the program achieving? What are the components getting delivered, are there any adaptations?	*Treatment adherence*	Descriptors of treatment components and methods of delivery	Purpose-developed questions							X			
	Is the service acceptable, appropriate and feasible from the perspectives of young people, support persons and providers?	*Acceptability*	Proportions of users and providers ratings	AAF Open-ended questions	X	X	X	X	X	X				
Effectiveness	For young people, does engaging with the service lead to: - Reduced self-reported self-harm, suicide ideation and attempts - Reduced re-presentations to ED - Improved mental health related to depressive symptoms and psychological distress? - Increased treatment engagement, social/vocational functioning and quality of life?	*Effectiveness*	Significant reduction from baseline to posttreatment on frequency and severity of suicidal ideations (primary outcome), and frequency and severity of self-harm and suicide attempts	SIDAS YRBS Self-harm (purpose-developed questions)	X		X							
			Significant reduction in hospital (re-)presentation trends to ED since the implementation of HOPE aftercare	Hospital (re)presentation rates (Surveillance data)										X
		*Improved mental health*	Significant improvement on mental health indicators	PHQ–9 GAD–7 K10 PSS	X		X							
		*Better functioning*	Treatment completion and dropout rates Significant improvement in functioning and quality of life	CHU–9D	X	X					X		X	
	Is the service safe?	*Safety*	Significant deterioration of symptoms Occurrence of serious adverse events	SIDAS, suicide attempt or hospital admission	X								X	
	Is the service acceptable, appropriate and feasible from the perspectives of young people, support persons and providers?	*Acceptability*	Proportion of service users and providers	AAF	X		X				X			
	Is the service cost-effective?	*Cost-effectiveness*	Program costs and costs of additional healthcare services (i.e., hospital presentations and psychological treatment) relative to quality of adjusted life years.	Hospital re-presentation RUQ CHU–9D	X								X	X
Maintenance	What are the long-term effects of the service on individual outcomes?	*Effectiveness (at follow-up)*	Significant further reduction or maintenance of symptoms at follow-up on measures of intensity and severity of suicidal ideations (primary outcome) and suicide attempt, self-harm, and mental health indicators and quality of life	SIDAS YRBS Self-harm (purpose developed questions) PHQ–9 GAD–7 K10 CHU–9D	X								X	X
	What are the likely impacts of the service when delivered at scale and within the current service eco-system?	*Scalability*	Computer-based system dynamics model											X

*EOT form *routinely collected end-of-treatment form, *ROM *Routine Outcome Measure, *Hospital* patient’s electronic medical records, *SHMS *Self-Harm Monitoring System for Victoria, *CFIR–IS *Consolidated Framework for Implementation Research Inner Setting measures [28], *ILS *Implementation Leadership Scale [29], *PSI *Program Sustainability Index [30], *ASP *Attitudes to Suicide Prevention Scale [31], *AAF *Acceptability, Appropriateness and Feasibility measures [32], *SIDAS *Suicidal Ideation Attributes Scale [33], *YRBS *United States Centers for Disease Control and Prevention’s National Youth Risk Behavior Survey [34], *PHQ–9 *Patient Health Questionnaire–9 [35], *GAD–7 *Generalized Anxiety Disorder Scale [36], *K10 *Kessler Psychological Distress Scale [37], *CHU–9D *Child Health Utility–9D [38, 39], *RUQ * Resource Use Questionnaire [40]

## Intervention

The Orygen HOPE aftercare is an evidence-informed, psychosocial program that provides intensive, person-centered, multi-disciplinary coordinated support which is tailored to the unique needs and circumstances of the individual young person using Relational Clinical Care (RCC) (see Table 2).

**Table 2:** HOPE aftercare core RCC components

The core components of RCC		
Collaborative formulation, diagnosis, and psychoeducation		
Establishing and attending to the therapeutic relationship with young person and carers to enhance engagement and reduce barriers to treatment		
Family, carer, and system inclusive practice		
Young person and carer lived-experience peer support		
Brief intervention and time-limited episodic care		
Focus on the episode of care with a shared understanding of endings and supported transitions		
Collaborative care planning, including:		
a) Identification of co-occurring difficulties contributing to the acute presentation b) Goal setting c) Shared decision making and formulated treatment planning.		
Consistency and containment within the HOPE team via supervision and reflective clinical discussions.		

Young people referred to the Orygen HOPE aftercare are contacted within 24-hours of hospital/ED discharge; in-person assessment is arranged within 72-hours of the initial referral, and treatment is provided for up to three months. The RCC model positions the young person as the leader of the care team, and the multidisciplinary team delivers holistic support across psychological, family, psychosocial, and physical domains. The main feature of the HOPE service is collaborative work with the young person’s support system (family/carers, educational/vocational, healthcare, and community support providers) to understand, respond to and meet the wellbeing needs of the young person.

Young people referred to the Orygen HOPE aftercare will receive an assessment and treatment under the care of the Orygen HOPE multidisciplinary team. This consists of a consultant psychiatrist, psychiatric registrar, and allied health staff (clinical psychologist, psychologists, social workers, occupational therapists) providing clinical services. Youth peer workers and family peer workers are employed to provide additional support services. Orygen HOPE aftercare is governed directly by a lead consultant psychiatrist and team coordinator and sits within the broader governance structures of Orygen clinical specialist programs.

### Participants

#### Young people and carers

All young people (aged 12–25) who receive care from the Orygen HOPE aftercare will be invited to participate in the evaluation. Recruitment of participants will be facilitated by the Orygen HOPE clinical team. Following admission to Orygen HOPE aftercare, Orygen HOPE clients are presented at the clinical team meetings for discussion and allocation. A research team member will attend these meetings to be informed about the admission of new clients and to determine when to approach potential participants. Family members, support persons, and carers will also be invited to participate. To be eligible for the service, young people must meet the following criteria:

- ▪ Age between 12 and 25 years.
- ▪ Presented to EDs within western and north–western metropolitan Melbourne following suicide attempt, or an episode of severe self-harm and/or significant levels of suicidal ideation.

Young people will be excluded from the service and referred to other appropriate services (e.g., other Orygen clinical services), if they present with any of the following criteria:

- ▪ Currently engaged in case management.
-  ▪ Require acute care (will be initially managed by Orygen Acute).
- ▪ Under a compulsory treatment order.
- ▪ Present with psychotic symptoms or symptoms of hypomania or mania.
- ▪ Have a complexity of mental health and psychosocial needs indicating a need for longer term case management or intensive care.

#### Service providers

All service providers involved in intake or delivery of the HOPE service will be invited to participate. This will include service coordinators, clinical leaders, clinicians, consultant psychiatrist, psychiatric registrar, youth peer support workers, family support workers, and Orygen’s intake assessors.

### Sample size and power

Based on a similar aftercare service developed for adults [22], we expect to detect a medium effect size of suicide ideation frequency reduction at the follow-up time point. To detect this effect, and assuming a correlation between baseline and follow-up measures of 0.5 and a drop-out rate of 30%, a sample of 184 young people will be needed.

### Data collection

To facilitate the evaluation, a range of data collection strategies and sources will be used, including electronic hospital records, self-report data, and surveillance and monitoring data (see Table 1).

Epidemiological data on rates of self-harm and suicidal ideation presentations across the catchment area will be obtained from routinely collected data extracted from electronic patient medical records. These data will be collected every six months throughout the life of the project and will provide information relevant to patients’ demographic characteristics, hospital presentations, referral sources, clinical descriptors, and assessments.

Patient-reported data will include data collected directly from young people and carers/parents/family members. Young people and family members will be recruited and consented to participation by the research team at the beginning of the young person’s treatment episode. Young people will provide quantitative survey responses at Time 1 (Baseline: at intake into treatment), Time 2 (Posttreatment: at discharge from treatment), and Time 3 (Follow-up: 3-months post–discharge from treatment). Family members/carers will provide quantitative survey responses at Time 1 and Time 2. Semi-structured interviews will be undertaken following completion of treatment with a select group of young people and family members/carers (*N* = 6–12 participants in each group). Interviews will be conducted in person or via video conference. The interviews will be conducted in Year 2 and Year 4 of this evaluation, allowing the service an opportunity to mature.

Service provider reported data will be collected via self-report surveys, individual semi-structured interviews, and group discussion via workshops, which will be undertaken annually. Specifically, surveys and interviews will be used to assess barriers and facilitators of the implementation across the five years. Workshops will be scheduled annually with the whole group of service providers and facilitated by the research team. The workshop discussion allows for appraisal of service strengths and weaknesses, and identification/prioritization of areas that need improvement. Using this methodology, providers identify the changes individually (via surveys) and then discuss responses in a participatory workshop to identify what needs to be improved to sustain the service.

Surveillance data will be collected from the Orygen’s Self-Harm Monitoring System for Victoria [28]. This database has been collecting information pertaining to self-harm and suicide ideation-related presentations across all major public EDs in Melbourne, Victoria.

### Measures

Demographic data (e.g., date of birth, gender, sex assigned at birth, country of birth) will be collected from participating young people and family members via surveys and from hospital-based records. Provider characteristics will be collected from providers via surveys, and will include HOPE aftercare role, time in role, total years’ experience in working with young people at risk of suicide, formal suicide-specific training received, and perception of training adequacy.

#### Primary outcome

*Suicidal ideation* will be measured using the Suicidal Ideation Attributes Scale (SIDAS; [29]). The SIDAS consists of five items (rated 0–10) each targeting an attribute of suicidal thoughts over the past month: frequency, controllability, closeness to attempt, level of distress associated with the thoughts, and impact on daily functioning. The SIDAS has demonstrated strong internal consistency and good convergent validity in a large online sample of Australian adults, including young people aged 18 and above [29].

#### Secondary outcomes

*Suicide attempts* will be assessed using four items from the United States Centers for Disease Control and Prevention’s National Youth Risk Behavior Survey (YRBS; [30]) assessing frequency and severity of suicide attempts over the past month. The YRBS displays appropriate psychometric properties for use with young people aged 13 to 18, including test–retest reliability [30].

*Self-harm* will be assessed using purpose-developed questions assessing type and frequency of self-harm events in the past month (e.g., “Have you ever engaged in an act of self-harm (with or without suicidal intent)? If yes, have you engaged in this behavior in the last month?”).

*Depressive symptoms* will be assessed using the 9-item Patient Health Questionnaire–9 (PHQ–9; [31]). The PHQ–9 focuses on severity of symptoms experienced over the past two weeks, with items rated on a 4-point Likert scale. It has established reliability and validity in acute and primary care [31], including young people [32, 33].

*Anxiety symptoms* will be assessed using the 7-item Generalized Anxiety Disorder Scale (GAD–7; [34]). The GAD–7 focuses on the frequency of symptoms experienced over the past two weeks, with items rated on a 4-point Likert scale. The GAD–7 has established reliability and validity in acute and primary care [31], including young people [35, 36].

*Psychological distress* will be assessed using the 10-item Kessler Psychological Distress Scale (K10; [37]). The K10 assesses generalized psychological distress experienced over the preceding 30 days. Items are rated on a 5-point Likert scale. The K10 has established reliability and validity across a variety of samples [37], including the Australian general population [38] and Australian adolescents [39].

*Mental health resource use* will be assessed using items adapted from the Young Mind Matters Service Use Questionnaire [40], referred to as the Resource Use Questionnaire (RUQ) for the purposes of the proposed study. These items assess relevant resource use (e.g., mental health services, ED attendance).

*Quality of life* will be assessed using the Child Health Utility–9D (CHU-9D) measure [41, 42]. The CHU–9D is a generic preference-based instrument designed to assess quality of life and facilitate economic evaluation of preventative and healthcare treatment programs aimed at young people. The CHU–9D has demonstrated practicality, face, and construct validity within a sample of Australian adolescents [43].

*The acceptability*,* appropriateness and feasibility* of the HOPE service will be measured using the Acceptability, Appropriateness, and Feasibility Measures (AAF) [44]. Items are rated on a 5-point Likert scale and assess the extent to which a given intervention is perceived as acceptable, suitable, and capable of being successfully implemented. The AAF has demonstrated appropriate psychometric properties [44].

Young people and their family members’/carers’ *satisfaction with the service* will be measured using routinely collected survey responses that gather information on domains including the degree to which service users feel their autonomy and values were respected, and their needs were supported during their episode of care.

*Treatment completion* including frequency, duration and components delivered during each individual episode of care, plus referral options provided following aftercare, will be collected via purpose-developed questions reported by providers via a short end-of-treatment form.

*Parental self-efficacy*, or confidence, around ability to engage in activities to support the young person in navigating a suicidal crisis will be measured using the 9-item Parental Self-Efficacy Scale (PSS; [45]). Items are rated on an 11-point scale. The PSS has reported good internal consistency [45].

*Provider knowledge and self-efficacy* will be assessed using purpose-developed questions based on previous program evaluations of suicide prevention (e.g., Zero Suicide) [46]. These questions will provide an overview of provider characteristics relevant to screening and assessing individuals for suicide risk (e.g., “I am comfortable screening individuals for suicide risk”), and providing appropriate care (e.g., “I am comfortable providing care to individuals who have been identified as being at elevated risk for suicide”), rated on a 5-point scale.

*Provider attitudes* will be assessed using the 14-item Attitudes to Suicide Prevention Scale (ASP; [47]). Items are rated on a 5-point scale. The ASP has demonstrated adequate reliability and internal consistency [47].

*Implementation moderators* will be measured using the Consolidated Framework for Implementation Research (CFIR) [48] Inner Setting measures (CFIR–IS; [49]). The CFIR–IS assesses culture overall, culture stress, culture effort, implementation climate, learning climate, leadership engagement, and available resources. The CFIR–IS has demonstrated appropriate psychometric properties [49].

*Implementation leadership* will be measured using the 12-item Implementation Leadership Scale (ILS) [50] supervisor and staff versions. The ILS comprises four subscales representing proactive, knowledgeable, supportive, and perseverant leadership. It has evidenced excellent internal consistency, as well as convergent and discriminant validity [50].

*Program sustainability* will be assessed using the 29-item Program Sustainability Index (PSI) [51]. The PSI assesses six elements: leadership competence, effective collaboration, understanding the community, demonstrating program results, strategic funding, staff involvement and integration, and program responsivity. The PSI has demonstrated acceptable internal consistency and validity [51].

The *Victorian Self-Harm Monitoring System* [28] will provide data regarding young people’s (12–25 years) presentations to EDs in the catchment area (north–west region of Melbourne) for the period between 1 January 2012 and 31 December 2026 (i.e., since the start of monitoring until the end of the evaluation). Data extracted will capture and compare hospital presentation trends across the specific time-period prior to HOPE service commencement (from 1 January 2012 to 31 December 2021) and following HOPE service commencement (from 1 January 2022 to 31 December 2026). Specifically, data extracted will utilize demographic descriptions (e.g., age, gender, postcode), rates of presentations for self-harm, clinical, and treatment characteristics (e.g., self-harm method, length of stay, discharge information, mental health referral).

#### Economic evaluation

Quantitative data obtained from the user surveys will inform the economic evaluation. The evaluation will involve collation of program delivery costs, other costs incurred or saved (e.g., reductions in hospitalizations or increases in psychological treatment) relative to the health benefits, including quality adjusted life years. The data sources described above (i.e., Victorian Self-Harm Monitoring System data) will contain some of the information required (e.g., rates of suicide attempts pre– and post– the introduction of the aftercare service), but there may still be some missing information, in particular, comprehensive assessment of service use in the absence of the aftercare service. The research team will explore different types of comparators based on key and supplementary questions, evidence from the literature, administrative data, expert opinion, and other relevant available datasets.

#### Study team logging procedures

To accommodate the study’s mixed method approach, study team logging procedures will be captured and described. These will relate to activities during any of the following: (1) the implementation or delivery of the service (e.g., changes in service staffing profile) (2), evaluation data collection processes (e.g., provider workshop discussions), and (3) regular procedures (e.g., meetings between the research and clinical team). As part of the study’s mixed method assessment procedures, the types, content, and frequency of these activities will be captured in logs, meeting minutes and field notes, and will be used to inform the evaluation results. Where necessary, desktop document audits will capture any broader policy or service changes.

#### Semi-structured interviews with users and providers

After the completion of the intervention, one-on-one semi-structured interviews will be conducted with a select group of young people and their family members. The interviews will explore satisfaction and acceptability of the service, and barriers and facilitators of participation and engagement with the service.

Annual one-on-one semi-structured interviews will be conducted with all service providers to accommodate staffing changes. These interviews will explore satisfaction with the service, as well as barriers and facilitators to its adoption and implementation.

### Data storage

There are several mechanisms in place to ensure confidentiality and privacy of the data. Research undertaken in this project will adhere to the requirements of State and National Privacy Principles (Privacy and Data Protection Act 2014; Privacy Act 1988 (Cth)), Health Privacy Principles (Victorian Health Records Act 2001), the Information Privacy Principles (Privacy and Data Protection Act 2014), and the National Health and Medical Research Council Act 1992.

As such, all data collected from participants for this study’s purpose will be safely stored in password protected databases on Orygen servers. Any paper-based data will be stored in secure and locked cabinets onsite at Orygen. All unique identifiers will be removed from the electronic datasets and confidentiality ensured by data custodians. Moreover, the data will not be re-identifiable by the researchers and all results will be reported in aggregate form, with no information presented that could be used to identify any individuals.

### Data analysis plan

#### Quantitative analyses

The primary objective of the statistical analyses will be to examine and compare changes in suicidal ideation over three time periods, from pre–treatment to post–treatment and to follow-up. This analytic approach will be replicated for all secondary outcomes (i.e., self-harm, suicide attempts, depression, anxiety, psychological distress, and quality of life). For continuous outcomes, changes in each outcome measure over time will be assessed using a linear mixed effects model. Binary outcomes will be modelled using a population-averaged generalized estimating equation (GEE), and sensitivity analyses will be undertaken using generalized linear mixed models. Path analysis will be used to explore the mediating effects on effectiveness of the intervention. Wherever possible, analyses will be conducted using intent-to-treat methods.

Data from the Self-Harm Monitoring System for Victoria will be used to model changes in rates of presentations, as well as changes in time to re-presentation to ED for self-harm and suicidal ideation, while also describing the differences in clinical characteristics (e.g., length of stay in the ED) and disposition characteristics (e.g., discharge destination). Analyses will include negative binomial regression and recurrent event survival models controlling for individual level risk factors as well as spatial and temporal risk factors [52]. Models will also be adjusted for autocorrelation between repeated events to protect against inflation of the type I error rate [52]. Data analysis will compare the period prior to HOPE service commencement (from 1 January 2012 to 31 December 2021) and following HOPE service commencement (1 January 2022 to 31 December 2026). Further analyses will also consider potential influence of the cohort trends between cohort groups (existing suicide/self-harm cohort trends), COVID–19 impact (cohort years 2019–2021), and other domain-specific confounders.

To assess the evaluation objectives set out in the second aim, data pertaining to the implementation outcomes will be described, and where possible identify core treatment components and adaptations as well as contextual factors impacting implementation and effectiveness of the service. For continuous outcomes, descriptive statistics will be used to assess each of the outcomes of interest, and changes over time will be examined where possible.

#### Economic evaluation

The economic evaluation will assess cost-effectiveness of the service or the value for money credentials using data described in the previous section. The long-term cost-effectiveness of the service will be determined using an economic evaluation suicide prevention model [53], which adopts a longer-term time horizon and estimates the reduction in suicide deaths using suicide attempts as a surrogate outcome.

#### Dynamic systems modeling

Dynamic systems model development will leverage project data, a range of administrative datasets, and research evidence to deliver an interactive decision support tool that provides a safe virtual environment to explore the optimal timing, scale and intensity of aftercare service needed to achieve the greatest population-level impacts within the contextual, resource and capacity constraints of the Victorian and national service systems, helping to guide optimal implementation at scale. Parameter values that cannot be derived from available data will be estimated via constrained optimization using Powell’s method. The model will be validated by testing whether the outputs of the model can replicate time series data across a range of key indicators (e.g., the prevalence of psychological distress, psychiatric hospitalizations, mental health-related ED presentations, self-harm hospitalizations, and suicide deaths). The process of system dynamics model development and typical data sources used has been reported in detail elsewhere [54].

The model will prospectively evaluate the population-level impacts of scaling up the aftercare service across the region under contextually relevant projected trajectories of suicidal behavior. A set of scenarios will be simulated to explore its projected impact and provide vital insights into how best to achieve optimal impact ahead of large-scale implementation. We will also explore the projected impact of combining the aftercare service with other acute care services in ways that may be synergistic in reducing suicide and/or self-harm related ED presentations. Sensitivity analyses will be performed to assess the impact of uncertainty in estimates of the direct effects of each intervention on the simulation results. Differences in projected outcomes between the baseline and intervention scenarios will be calculated for each set of parameter values and summarized using simple descriptive statistics. Model construction and analysis will be performed using Stella Architect (version 3.4).

#### Qualitative analysis

Qualitative data will be analyzed using an applied thematic analysis approach [55] and will involve the following steps: (1) understanding the data; (2) generating initial codes; (3) generating themes; (4) reviewing themes; (5) refining and naming themes; (6) producing findings. Results will be presented as thematic counts, vignettes and/or tabulated representation of themes with illustrative quotes. The implementation framework will be used to understand and interpret the findings.

### Mixed methods analysis

Qualitative data will be converged with the quantitative data in a mixed methods design. This will involve triangulation of qualitative and quantitative data either [1] *simultaneously* to answer the same questions (QUAN + QUAL), i.e., simultaneous use of one data to validate conclusions reached from analysis of the other data, or [2] *sequentially* to confirm the same answer (QUAN ◊ qual), i.e., sequential use of one dataset to answer questions raised from analysis of the other dataset. This design taxonomy will therefore use qualitative data to expand upon the results of the quantitative data to understand the implementation processes as reported by stakeholders [56].

## Discussion

This evaluation protocol responds to the rising rates of youth suicide in Australia, and internationally. Successful prevention advocacy has led to the widespread roll-out of aftercare services (e.g., [16]). These services are designed to support adults who have presented to the ED with self-harm/suicide risk, and are currently under evaluation [22, 57]. This evaluation will contribute to these joint evaluation efforts, by providing the first-ever evaluation of a youth-focused aftercare service implemented in public healthcare in Australia.

This evaluation also responds to national and international calls for better evaluations of suicide prevention interventions implemented in complex settings [58]. This evaluation will respond to these calls by providing a rigorous and comprehensive evaluation encompassing evaluation and implementation conceptual frameworks to answer key research questions regarding effectiveness, cost-effectiveness, and scalability of the youth-focused aftercare service. The proposed evaluation is the first-ever application of hybrid type I design in suicide prevention evaluation research. The key advantage of the hybrid study design is its capacity to establish and expand the evidence base regarding effectiveness of the intervention while considering the context for implementation. The use of the evaluation and implementation frameworks to guide data collection and analysis will provide a comprehensive approach to assessing, reporting, and interpreting the findings. A mixed methods approach—which capitalizes on multiple data sources—improves efficiency and ensures consideration of themes and findings outside of primary reports provided via quantitative data. This will reveal great insights about service user and provider experiences and reinforce validity and inform the continuous implementation. The use of economic evaluation is critical and will help bridge the research-to-practice gap and inform implementation strategy. Simulation modelling plays a pivotal role informing the scale-up initiatives and sustainability planning.

Through rigorous methodology and consistent engagement at every step of the project from initial intervention co-design, to service implementation, evaluation planning and governance, this evaluation will continue to integrate cumulative knowledge, perspectives, experiences, and objectives of all stakeholders, including young people and their families, providers, researchers, and policy makers. Together the methods outlined in this protocol will generate critical new knowledge that will feed back into research, provide decision makers with vital information to guide implementation decisions and roll-out of similar suicide prevention services nationally and internationally.

## Data Availability

Not applicable, as this manuscript does not contain any data or materials.
